# ﻿*Thesiummuasyae* (Santalaceae), a new species from the limestone fynbos of the Overberg, South Africa

**DOI:** 10.3897/phytokeys.201.80774

**Published:** 2022-06-16

**Authors:** Daniel A. Zhigila, A. Muthama Muasya

**Affiliations:** 1 Bolus Herbarium, Department of Biological Sciences, University of Cape Town, Rhodes Gift 7707, Cape Town, South Africa University of Cape Town Cape Town South Africa; 2 Botany Programme, Department of Biological Sciences, Gombe State University, PMB 127, Tudun Wada, Gombe, Gombe State, Nigeria Gombe State University Gombe Nigeria

**Keywords:** Endemic, Greater Cape Floristic Region, systematics, taxonomy, Thesiaceae

## Abstract

*Thesiummuasyae*, a new species of the family Santalaceae, is described and illustrated. This species has unique morphological and ecological characters, differentiating it from other closely related species of the genus, such as *T.karooicum*. These characters include plants forming compact shrubs to about 30 cm tall with glabrous surfaces; leaves recurved, to about 4 cm long, terete to triangular, apiculate; flowers placed in lax spikes or borne solitary; and style up to about 2.5 mm long. Ecologically, *T.muasyae* is endemic to the limestone fynbos in the Overberg, Bredasdorp District, South Africa. Molecular phylogenetic evidence places this species in Subgenus FriseaSectionBarbata, as closest sister to *T.hispidulum* + *T.karooicum*. A preliminary conservation Red List assessment suggests that *T.muasyae* is Critically Endangered, based on its population size, area of occupancy and extent of occurrence.

## ﻿Introduction

*Thesium* L. is the largest genus in the family Santalaceae with > 360 species (the Angiosperm Phylogeny Group IV 2016; POWO 2022). Species in this genus are root hemi-parasites found in Africa, Asia, Australia, Europe, South America and by introduction, North America (Nickrent et al. 2010; García et al. 2018; Zhigila et al. 2020; POWO 2022). The Cape of South Africa is its origin and centre of diversity (Pilger 1935; Moore et al. 2010). *Thesium* species are found in several biomes and is abundant in the Fynbos (especially in fynbos and renosterveld vegetation types), Albany Thicket, and Succulent Karoo (Hill 1925; Manning and Goldblatt 2012). The species inhabit different substrates but are mainly found on areas with sandstone, quartz, shale, deep coastal sand and limestone (Manning and Goldblatt 2012). Species in the genus exhibit diverse growth habits including erect, suberect to prostrating herbs, rhizomatous shrublets under 10 cm tall and shrubs to small trees to about 2 m tall (Hill 1925). The genus is diagnosed by a combination of complex morphological characters such as leaves without distinct petioles that are adpressed to the stems, flowers usually with external glands between the perianth lobes, ovaries with the placental column twisted or straight, and fruits indehiscent nutlets with prominent veins and persistent perianth segments (Hill 1915; García et al. 2018).

Recent molecular phylogenetic analyses for Santalaceae, with robust taxa and loci sampling of *Thesium*, supported a monophyletic genus (Moore et al. 2010; García et al. 2018; Zhigila et al. 2020). The sampling comprised all of the Greater Cape Floristic Region (GCFR) species and worldwide representatives of *Thesium* using four plastid (*trnL-F*, *matK*, *rpl32-trnL* and *rbcL*) and one nuclear (ITS) DNA regions. Based on this well-developed phylogeny, Zhigila et al. (2020) confirmed the monophyly of *Thesium* and hypothesized five subgenera within the genus namely *Hagnothesium* (A.DC.) Zhigila, Verboom and Muasya, *Thesium* L., *Discothesium* (A.DC.) Zhigila, Verboom and Muasya, *Psilothesium* (A.DC.) Zhigila, Verboom and Muasya and *Frisea* (Rchb.) Hendrych, with the subgenus Frisea having the highest number of species (103 species, most of which are South African) and *Hagnothesium* being endemic to the GCFR. Within the GCFR, molecular data revealed genetic variations for some taxa that may represent different species. These data have spurred our interest for further field surveys in the botanically rich but poorly explored renosterveld and limestone fynbos patches of the Overberg.

Eleven species are currently recorded in the limestone fynbos and renosterveld of the Overberg region, South Africa (Curtis-Scott et al. 2020). Five of them, *T.dmmagiae* Zhigila, Verboom and Muasya, *T.nigroperiathum* Zhigila, Verboom and Muasya, *T.quartzicolum* Zhigila, Verboom and Muasya, *T.rhizomatum* Zhigila, Verboom and Muasya and *T.stirtonii* Zhigila, Verboom and Muasya are endemics (Zhigila et al. 2019a). Except for *T.quartzicolum* and *T.stirtonii* (found on quartz outcrops of the Overberg), these species are confined to the shale scrubs or ecotones of shale and limestone slopes south east of the Vanderstalkraal Private Farm. In this paper, we describe a fifth species endemic to the limestone outcrops of the Overberg in the GCFR. The illustrations, distribution, molecular phylogenetic relationships and preliminary conservation status are presented. This work forms part of the series of the published works on the GCFR*Thesium* species (Zhigila et al. 2019a, 2019b, 2020) as well as the larger project to revise the entire genus (see Mashego and le Roux 2018; Visser et al. 2018; Lombard et al. 2021).

## ﻿Materials and methods

### ﻿Morphological assessments

The morphological assessments of the new species were carried out on our field collections and on herbarium specimens deposited at BOL, NBG (including SAM and STE vouchers) and PRE (codes as indicated by Thiers 2022), as well as online voucher materials (JSTOR 2022). Micromorphological characters were observed using a hand lens (10×) or under stereomicroscope Leica S9i with Nikon DS-5M Camera attached. The holotype of *T.muasyae* was deposited at BOL and duplicates distributed to NBG, PRE and K. Morphological terms were adopted from the recent *Thesium* taxonomic treatments of García et al. (2018), Zhigila et al. (2020) and Lombard et al. (2021).

### ﻿Molecular work and barcoding

Whole genome DNA was extracted from the silica-gel dried leaf materials collected during our fieldworks between 2019 and 2021. The extraction was performed using a modified CTAB protocol (Doyle and Doyle 1987) as amended by Zhigila et al. (2020). The following primers: ITS 4 (5´-TCC TCC GCT TAT TGA TAT GC-3´), ITS5 (5´-GGA AGT AAA AGT CGT AAC AAG G-3´) (White et al. 1990), trnL-C (5´-CGA AAT CGG TAG ACG CTA CG-3´) and trnL-F (5´-TT TGA ACT GGT GAC ACG AG-3´) were used to amplify and sequence the regions (Taberlet et al. 1991). The PCR mix per 30 μl reaction volume included 19.3 μl distilled H_2_O, 3 μl of 10× buffer, 1.25 μl MgCl_2_, 1.2 μl dNTP, 1 μl BSA, I μl DMSO, 0.9 ul each of forward and reverse primers, 0.3 μl kappa taq and 1.2 μl DNA template. For amplification, the PCR thermal condition included a 2-min initial denaturing step at 94 °C, then 30 cycles of 1 min denaturation, followed by annealing for 1 min at 50 °C, extension for 2 min at 72 °C, further extension for 7 min at 72 °C, and kept at 4 °C as amended by Zhigila et al. (2020). Sequencing reactions for both reverse and forward reactions were performed at the Stellenbosch University Sequencing Facility using the amplification primers.

Forward and reverse reaction sequences were assembled using Chromaspro version 2.1.5 (Technelysium 2017) and were aligned in MAFFT online service (Kuraku et al. 2013; Katoh et al. 2019). Each DNA locus was assessed and edited manually with the package BioEdit v. 7.2.6 (Hall 1999). The newly generated DNA sequences were deposited at the GenBank public repository with accession numbers OM746331–OM746335 for nrITS, and OM857946 and OM857954 for *trnL-F*. Tree files including sequences from previous studies were submitted to TreeBase (study number TB2:S24838) and are provided as Suppl. material 1.

A model-based Bayesian method (MrBayes) was used for the phylogenetic analyses on XSEDE v.3.2.6 (Ronquist et al. 2017) using the Cyber-Infrastructure for Phylogenetic Research (CIPRES) V.3.3 (Miller et al. 2010) platform. The GTR+G, since it is deemed to be the best-fit nucleotide substitution model with the Akaike and Bayesian Information Criterion, was selected as determined in jModelTest2 (Darriba et al. 2012). In two independent runs for the Markov Chain MCMC permutations, four simultaneous chains were initiated with a random tree run for 50^7^ generations with the trees sampled at every 10^3^ generations. Discarding burn-in trees of 25%, summaries of 50% majority-rule consensus trees were held. For Maximum Likelihood (ML) analyses, jModelTest2 selected GTR+G for nrITS, GTR + I + G for *trnL-F* and GTRGAMMA for combined dataset as the best-fitting models. The package RAxML v8 (Stamatakis 2014) was used for all analyses. Setting petitions for each region and the combined dataset with 1000 replicates of bootstrap analysis. In both BI and ML analyses, the posterior probabilities (PP) and percentage bootstrap support (BS) values respectively were used to indicate support for clades. The phylogenetic trees were visualised and edited in FigTree v1.4.4 (Rambaut 2018).

### ﻿Conservation assessments

The preliminary conservation Red List status for the species was determined using the IUCN guidelines (IUCN 2017). The extent of occurrence (EOO) and areas of occupancy (AOO) of the new species was assessed using the software Geospatial Conservation Assessment Tool (GeoCAT), with the default cell size of 2 × 2 km matrix (Bachman et al. 2011; GeoCAT 2021).

## ﻿Results

### ﻿Taxonomic treatment

#### ﻿ Thesium muasyae

Taxon classificationPlantaeColeopteraStaphylinidae

Zhigila sp. nov.

57CF72AF-A114-59B5-A96D-87214F415E17

urn:lsid:ipni.org:names:77299819-1

Figs 1 , 2

##### Type.

South Africa. Western Cape Province, Bredasdorp District, on limestone ridges, south east of Vanderstelskraal Farm, Overberg, 34°24'53.2"S, 20°15'10.5"E [34.41478°S, 20.25292°E]; elev. 60 m; 21 October 2021, *D.A. Zhigila & A.M. Muasya 1308* (holotype, BOL; isotypes: K, NBG, PRE).

##### Diagnosis.

*Thesiummuasyae* is morphologically similar to *T.karooicum*Compton (1931). Both species have robust woody habits, well-developed terete to triangular and imbricate leaves, elongated styles, conspicuous external glands between the perianth lobes, persistent perianth segments longer than the fruits, and elaiosomes (Table 1). However, *T.muasyae* differs from *T.karooicum* in its branching pattern being intricate to sympodial, stems and leaves glabrous, leaves recurved, flowers in lax elongated terminal spikes or racemes in leaf and bract axils, patelliform flowers with post-staminal trichomes attached to the anthers (versus branching pattern divaricate to virgate, stems and leaves minutely scabrous, leaves erect, flowers in terminal capitate head or clusters, urceolate flowers with post-staminal trichomes free from anthers in *T.karooicum*). Further the two do not overlap in distribution and ecology, *T.muasyae* is restricted to the Overberg limestone outcrops whereas *T.karooicum* is found on the Sandstone Mountains of the Succulent Karoo. *Thesiummuasyae* is also similar to *T.sonderianum*, but differs in the branching pattern being sympodial to intricate, plant surface glabrous, leaf apex apiculate, inflorescences solitary spikes on branchlets, perianth external glands present, stigma above the anthers, found on limestone slopes (versus dichotomously branched, plant surface minutely pubescent, leaf apex acutely mucronate, inflorescences terminal globose spikes, perianth external glands absent, stigma below the anthers, and restricted to the grasslands in *T.sonderianum*). Comparisons of important morphological characters of *T.muasyae*, *T.hispidulum*, *T.karooicum* and *T.sonderianum* are presented in Table 1.

**Table 1.** Main differentiating morphological features of *Thesiummuasyae* from its most-similar congeners.

	*T.muasyae*	*T.karooicum*	*T.sondarianum*	*T.hispidulum*
Plant height	10–30 cm	10–70 cm	50–100 cm	10–50 cm
Branching pattern	sympodial to intricate	divaricate	dichotomous	decumbent
Plant surface	Glabrous	minutely pubescent	minutely pubescent	pubescent
Leaf curvature	recurved or straight	recurved	recurved	recurved or straight
Leaf margin	Terete	scabrous	scabrous	scabrous
Leaf apex	Apiculate	acuminate	acutely mucronate	acuminate
Inflorescent type	elongated lax or solitary spikes	globose spikes	globose spike	globose spikes
External glands	present	present	absent	absent
Style length	1–2.5 mm	0.5–1.5 mm	1–2.5 mm	0.3–0.4 mm
Style	above anther	below anther	below anther	below anther
Anther	exserted	exserted	partly exserted	inserted
Post-staminal trichomes	attached to anthers	free from anthers	attached to anthers	attached to anthers
Fruit length	4–7 mm	4–10 mm	5–10 mm	3–4 mm
Fruit ribs	5-ribbed	10-ribbed	5-ribbed	10-ribbed
Substrate	limestone slopes	sandstone and shale	sandstone	sandstone and shale
Biome	Limestone Fynbos	Succulent Karoo	Grassland	Sandstone Fynbos

##### Description.

A perennial shrub, arising from woody rootstock, glabrous, to about 30 cm tall. ***Stems*** woody, erect to suberect, much branched, 3.0–5.0 mm in diameter, deeply grooved longitudinally. ***Branches*** 10–20 in number, mainly from the base, scarcely grooved, angled from > 45° to < 90°, branching pattern intricate to sympodial. ***Leaves*** terete to triangular, somewhat succulent, adpressed to the branchlets, lanceolate or oblanceolate or somewhat triangular, 1.5–3 × 0.5–1.5 cm, basally decurrent, midrib inconspicuous, not keeled but recurved, margins not distinct or entire, apically apiculate. ***Inflorescences*** a lax terminal spike or flowers solitary in leaf and bract axils. Bracts 2–4, leaf-like, slightly adnate to the base of peduncle, linear to lanceolate, 1.0–2.0 × 0.3–0.5 mm, margin entire, apex acute to acuminate, green; bracteoles bract-like, but smaller, adpressed to the pedicel, shorter than flower length. ***Flowers*** patelliform, on short peduncles, 5-merous, 2.0–5.5 × 1.5–5.0 mm, perianth lobe segments lanceolate, external gland conspicuously elongated between perianth lobe segments, 2.0–2.5 × 1.0–1.2 mm, lobe apex uncinate, obtuse, incurved, perianth lobe apical trichomes present, lobe margins entire, lobe internal colour white, external colour greenish black; hypanthium clearly marked, to about 0.5 mm long, hypanthium length longer than perianth lobe tube and wider. ***Stamens*** equal flower merosity, 0.2–0.3 mm long, staminal filaments exserted slightly above stigmas, attached to the perianth lobe walls by a tuft of trichomes, downwardly-directed basal trichomes absent. ***Style*** together with stigma 4–6 mm long; placental column twisted. ***Fruits*** subglobose to oblong, ovary portion oval, 5.0–8.0 × 4.5–5.5 mm, green to creamy green, glabrous with 10 conspicuous longitudinal ribs, reticulate veins prominent, pedicels enlarging into elaiosomes, persistent perianth segments equal to longer than the fruit.

**Figure 1.**
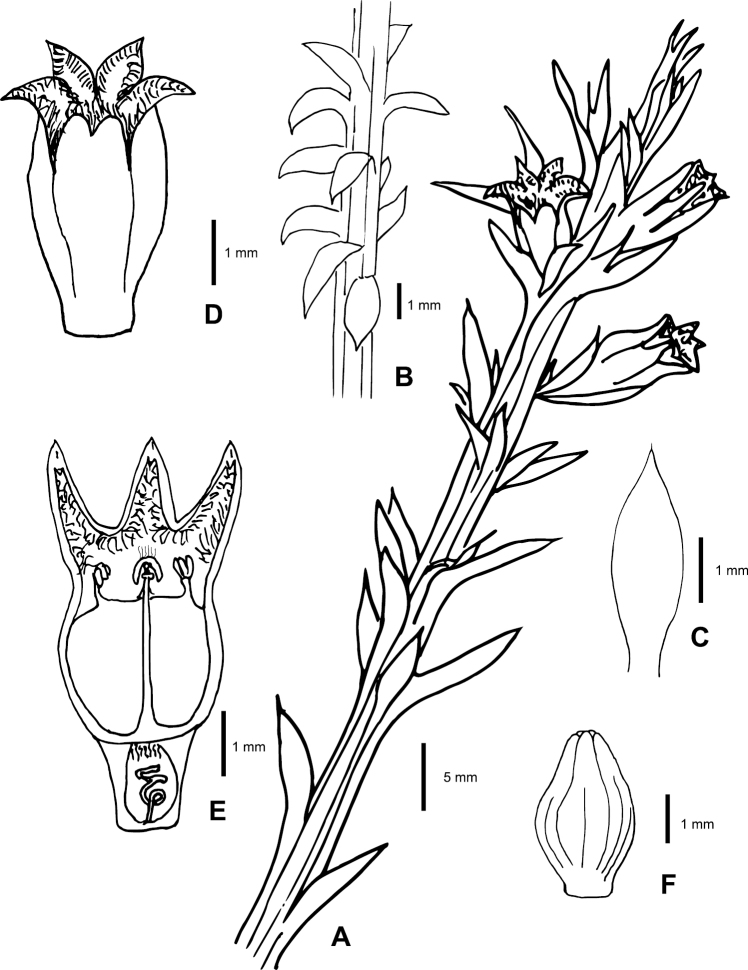
*Thesiummuasyae* sp. nov. **A** twig showing inflorescence arrangements **B** branchlet showing vegetative arrangements **C** bract **D** flower lateral view **E** flower dissected longitudinally **F** fruit. Line drawing by Pia M. Eibes.

**Figure 2.**
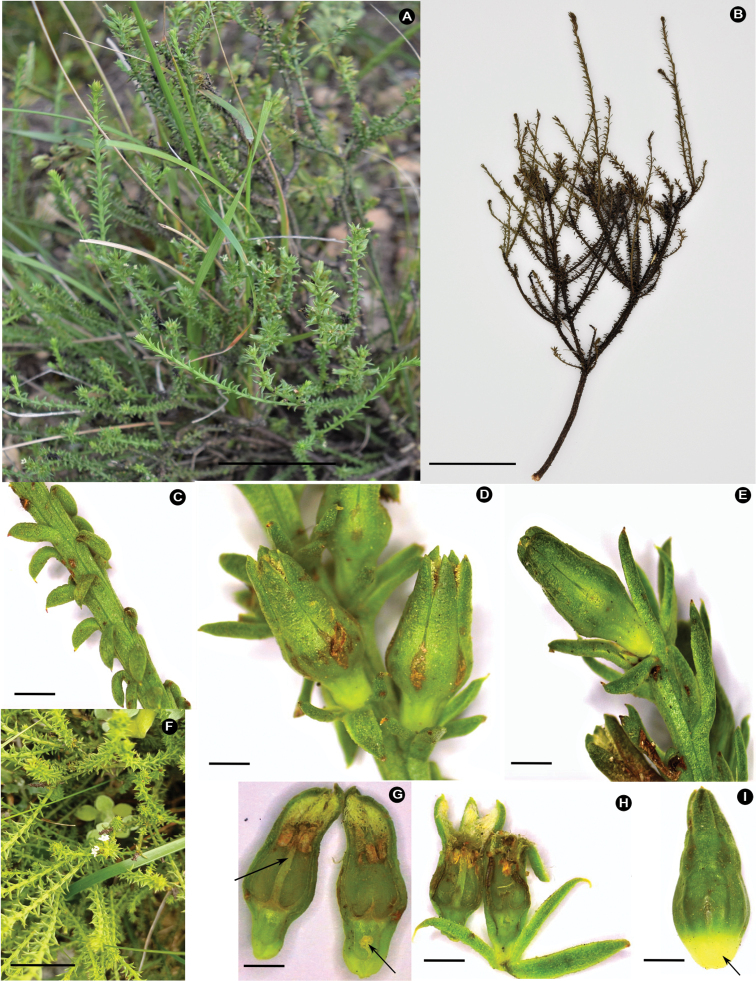
Morphological features of *Thesiummuasyae***A** whole plant in habitat **B** type material *D.A. Zhigila & A.M. Muasya 1308***C** branchlet and leaves **D** fruiting branchlet **E** fruit lateral view **F** inflorescences and leaves **G** flower longitudinal section showing long style in relation to anthers and twisted placental column **H** flower subtended by bracts **I** elaiosome on fruit. Photographs by Daniel A. Zhigila. Scale bars: 0.5 mm.

##### Distribution and ecology.

**Figure 3.**
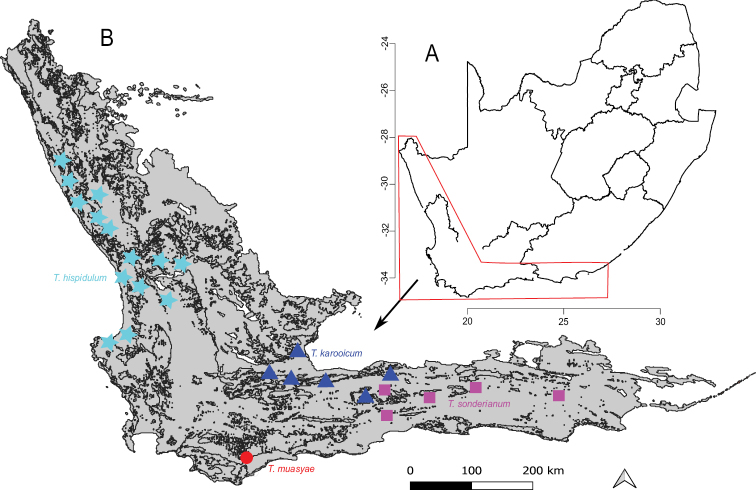
Map of **A** South Africa with the red outline indicating the Greater Cape Floristic Region (GCFR) **B** the GCFR showing the type locality (red solid circle) of *Thesiummuasyae* and of the congener species, *T.hispidulum* (aqua solid stars), *T.karooicum* (blue solid triangles) and *T.sonderianum* (fuchsia solid squares).

*Thesiummuasyae* was collected on the limestone ridges, south east of Vanderstelskraal Farm, Overberg, Bredasdorp District, Western Cape Province, South Africa (Fig. 3, triangles) at elevations less than 80 m above sea level. This species occurs on limestone and shale-limestone ecotone scrubs. The limestone soil in the type locality is characterised mainly by calcium carbonate, tiny fossils and other fossilized debris from the coastal limestone of the Bokkerveld Group (Finch et al. 2014; Penn-Clarke et al. 2018). Physically, the limestone soil is grey to whitish brown.

##### Phenology.

The collections were made in October with fruits and few flowers. Based on the average of 40 days from flowering to fruiting stage in *Thesium* species (pers. obs.), we can then extrapolate the flowering period to be between August and November.

##### Etymology.

The specific epithet ‘*muasyae*’ honors Professor A. Muthama Muasya for his immense contribution to the floristics and taxonomy of the Overberg and Cape plant species.

##### Conservation status.

We estimated a total of 10–20 individuals of *T.muasyae* in a single population over an extent of 0.0 km^2^ and the area of occupancy of about 5.0 km^2^. Although this species is on a private farm, grazing from livestock is an immediate threat. In addition, the entire Overberg Renosterveld habitat is considered as Endangered due to intense agricultural activities and the areas being fire-prone (von Staden 2015; Topp and Loos 2019). These threats together with the GeoCat geographical range estimations translate to the criterion B2, Critically Endangered category of the IUCN (2017) for *T.muasyae*.

##### Additional specimens examined.

South Africa. Western Cape Province, Bredasdorp District, on limestone ridge, south east of the Vanderstelskraal Farm, 34°24'52.1"S, 20°15'8.1"E [34.41447°S, 20.25225°E], elev. 63 m, 21 October 2021, *D.A Zhigila & A.M Muasya 1312* (BOL!); 34°24'53.2"S, 20°15'10.5"E [34.41478°S, 20.25292°E], elev. 65 m, *A.M Muasya & D.A Zhigila 8276* (BOL).

### ﻿Phylogenetic placement

**Figure 4.**
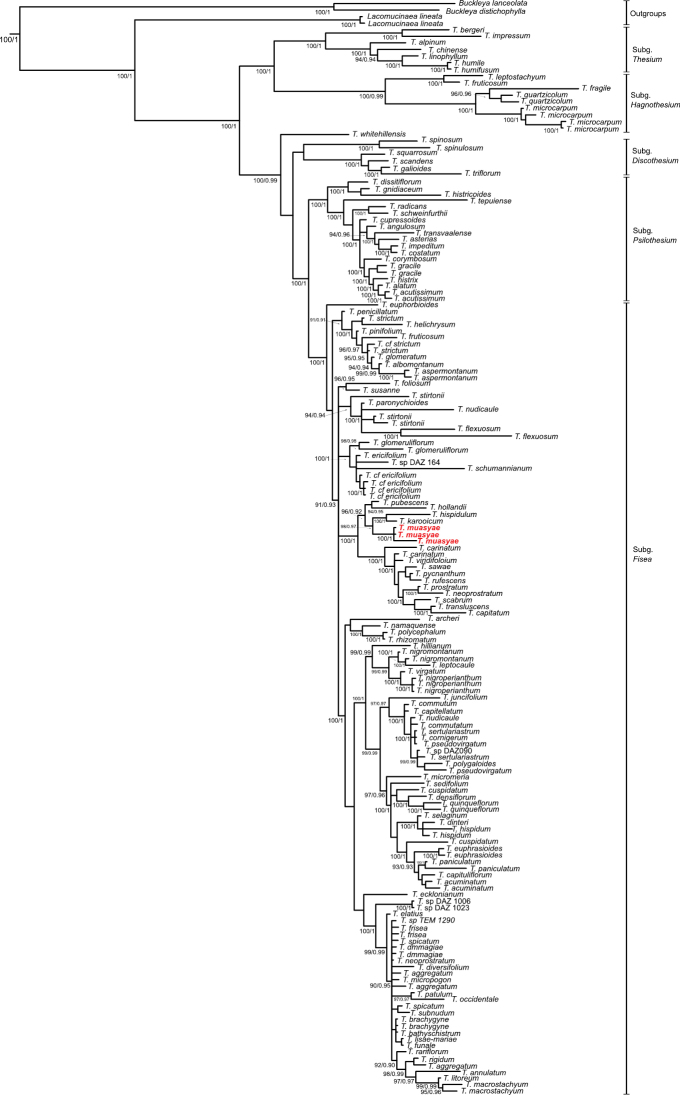
A 50% majority-rule consensus tree for Santalaceae that include the new species *Thesiummuasyae* (red bold) based on a combined nrITS and plastid *trnL-F* regions obtained from Bayesian Inference. Numbers on the nodes indicate clades with bootstrap and posterior probability support values of > 95% and 0.90 respectively.

The Maximum Parsimony and Bayesian analyses placed *T.muasyae* (red bold on Fig. 4) in a clade consisting of *T.karooicum*, *T.hollandii* and *T.hispidulum* with strong bootstrap and posterior probability values (BS = 100% and PP = 0.99 respectively). This clade is in the Subgenus Frisea, Section Barbata. The molecular placement supports the morphological similarities of *T.muasyae* and the congener species as stated in Table 1 and the diagnosis section above.

## ﻿Discussion

The morphological characters suggest that *T.muasyae* fits into section Barbata (Hill 1915), in the subgenus Frisea (Reichenbach 1828; Zhigila et al. 2020). Species in this leafy clade (sensu Moore et al. (2010)) share morphological characters such as stem transverse sections grooved, leafy stems (not scattered), linear to lanceolate leaves, determinate inflorescences, flowers having tuft of trichomes at perianth lobe apices, and flower shape patelliform, conspicuous external glands between perianth lobes, elongated perianth tubes and anthers attached to the tubes by post-staminal hairs and style 4–6 mm long (Hill 1915). However, *T.muasyae* differs from species in this clade in its growth height being < 30 cm tall (versus the typical 30–120 cm tall in *Barbata* clade), sympodial to intricate branching pattern (versus usually virgate or fastigiate branching patterns), leaves terete to triangular (versus leaves with distinct upper or lower surfaces to sometimes triangular), leaf apices apiculate (versus predominantly acute to acuminate), flower solitary, in leaf axils and terminal heads (versus raceme-like, cymose or globose spikes in most other *Barbata* species). The results of the molecular analyses are congruent with the previous studies (e.g. Moore et al. 2010; García et al. 2015; Zhigila et al. 2020) and support the morphological evidence to recognise *T.muasyae* as novel to science.

In the last five years, nine new species and several new records of *Thesium* have been discovered from the Overberg Region ([Bibr B37][Bibr B38]; Lombard et al. 2021). Most of these new taxa are evolutionary unique and having narrow ranges. For example, narrow-ranged and critically endangered *T.rhizomatum* and *T.nigroperiathum* are endemic to the limestone and ecotones of limestone fynbos and shale renosterveld of the type locality of *T.muasyae*. Hence, these new generic records have expanded our understanding of the biogeographic coverage and habitat diversity of species in the genus *Thesium*.

## Supplementary Material

XML Treatment for Thesium muasyae

## Supplementary materials

Supplementary material 1 Suplementary tree Data type (phyl. file) Explanation note Tree files including sequences from previous studies. https://binary.pensoft.net/file/702535This dataset is made available under the Open Database License (http://opendatacommons.org/licenses/odbl/1.0/). The Open Database License (ODbL) is a license agreement intended to allow users to freely share, modify, and use this Dataset while maintaining this same freedom for others, provided that the original source and author(s) are credited.Daniel A. Zhigila, A. Muthama Muasya
